# Diagnosis and treatment of “chronic Lyme”: *primum non nocere*

**DOI:** 10.1186/s12879-023-08618-w

**Published:** 2023-10-02

**Authors:** Prat Sébastien, Dalbin Jacques, Plotton Catherine, Gocko Xavier

**Affiliations:** 1 University of Clermont Auvergne, Auvergne, France; 2 Campus Santé Innovations, SAINT-PRIEST-EN-JAREZ, Jean-Monnet University, 10 RUE de la Marandière, 42270 Saint-Étienne, France

**Keywords:** Post-Lyme disease syndrome, Diagnostic errors, Overdiagnosis, Overtreatment, Adverse drug event

## Abstract

**Background:**

Approximately 10% of patients experience prolonged symptoms after Lyme disease. PTLDS (post treatment Lyme disease syndrome) is a controversial topic. It has been described as a source of overdiagnosis and off-label treatment. This review aims to describe the diagnostic errors and adverse events associated with the diagnosis and treatment of PTLDS.

**Methods:**

systematic review of the literature in the Medline and Cochrane Library databases, according to PRISMA criteria, including randomized clinical trials (RCT), observational studies, and case reports addressing diagnostic errors and adverse events published between January 2010 and November 2020 in English or French. Selection used a quadruple reading process on the basis of the titles and abstracts of the different articles, followed by a full reading.

**Results:**

17 studies were included: 1 RCT, 6 observational studies and 10 case reports. In the 6 observational studies, overdiagnosis rates were very high, ranging from 80 to 100%. The new diagnoses were often psychiatric, rheumatological and neurological. Disorders with somatic symptoms were often cited. Diagnostic delays were identified for cancers and frontoparietal dementia. In the RCT and observational studies, prolonged anti-infective treatments were also responsible for adverse events, with emergency room visits and/or hospitalization. The most common adverse events were diarrhea, sometimes with *Clostridium difficile* colitis, electrolyte abnormalities, sepsis, bacterial and fungal infections, and anaphylactic reactions.

**Conclusion:**

This review highlights the risks of prolonged anti-infective treatments that have not been proven to be beneficial in PTLDS. It emphasizes the ethical imperative of the “primum non nocere” principle, which underscores the importance of not causing harm to patients. Physicians should exercise caution in diagnosing PTLDS and consider the potential risks associated with off-label treatments.

## Background

Approximately 10% of patients experience prolonged symptoms (asthenia, diffuse pain, cognitive problems, etc.), after Lyme disease [1]. In 2006, the Infectious Diseases Society of America published a definition for post treatment Lyme disease syndrome (PTLDS). This definition relies on the development of significant fatigue, widespread musculoskeletal pain, and/or cognitive difficulties that last for a period of at least 6 months, and began within 6 months of Lyme diagnosis, and recommended treatment (standard of care antibiotics) [2]. PTLDS patients often experience a feeling of non-recognition and abandonment by physicians [3–5]. Faced with these feelings, they sometimes consult informally specialized doctors, who recommend the use of uncertified tests in private laboratories, and unapproved anti-infective drugs [6–8].

These off-label management issues raise the question of misdiagnosis and overdiagnosis of Lyme borreliosis (LB). These misdiagnoses could affect 9 out of 10 patients attributing their symptoms to LB [9–12]. Such overdiagnosis prompted the American College of Rheumatology (ACR) to include LB in the “Top five list” as part of the “Choosing Wisely” campaign [13].

These treatments also raise the question of the benefit-risk balance. Several randomized clinical trials have tested various anti-infectives in PTLDS. These studies did not show evidence of benefit from the treatments [14–18].

This review aims to describe the diagnostic errors and adverse events associated with the diagnosis and treatment of PTLDS.

## Methods

A review of the literature was conducted according to the PRISMA criteria.

### Inclusion criteria

Randomized clinical trials (RCTs), observational studies and case reports addressing diagnostic errors and adverse drug reactions in PTLDS published between January 1, 2010, and November 5, 2020, in French or English, were included. Articles were included regardless of patient gender or age.

### Exclusion criteria

Position papers or recommendations for PTLDS were excluded.

### Search equations and databases

With the help of a librarian, the Medline and Cochrane Library databases were searched with the following search equation: “Lyme disease” [MeSHTerms] OR “Lyme neuroborreliosis” [MeSHTerms] OR “erythema chronicum migrans” [MeSHTerms] OR “post Lyme disease syndrome” [MeSHTerms]) AND “inappropriate prescribing” [MeSHTerms] OR “diagnostic errors” [MeSHTerms] OR " [MeSHTerms] OR “adverse effects” [SH] OR “poisoning” [MeSHTerms].

### Selection of articles

**Fig. 1:**
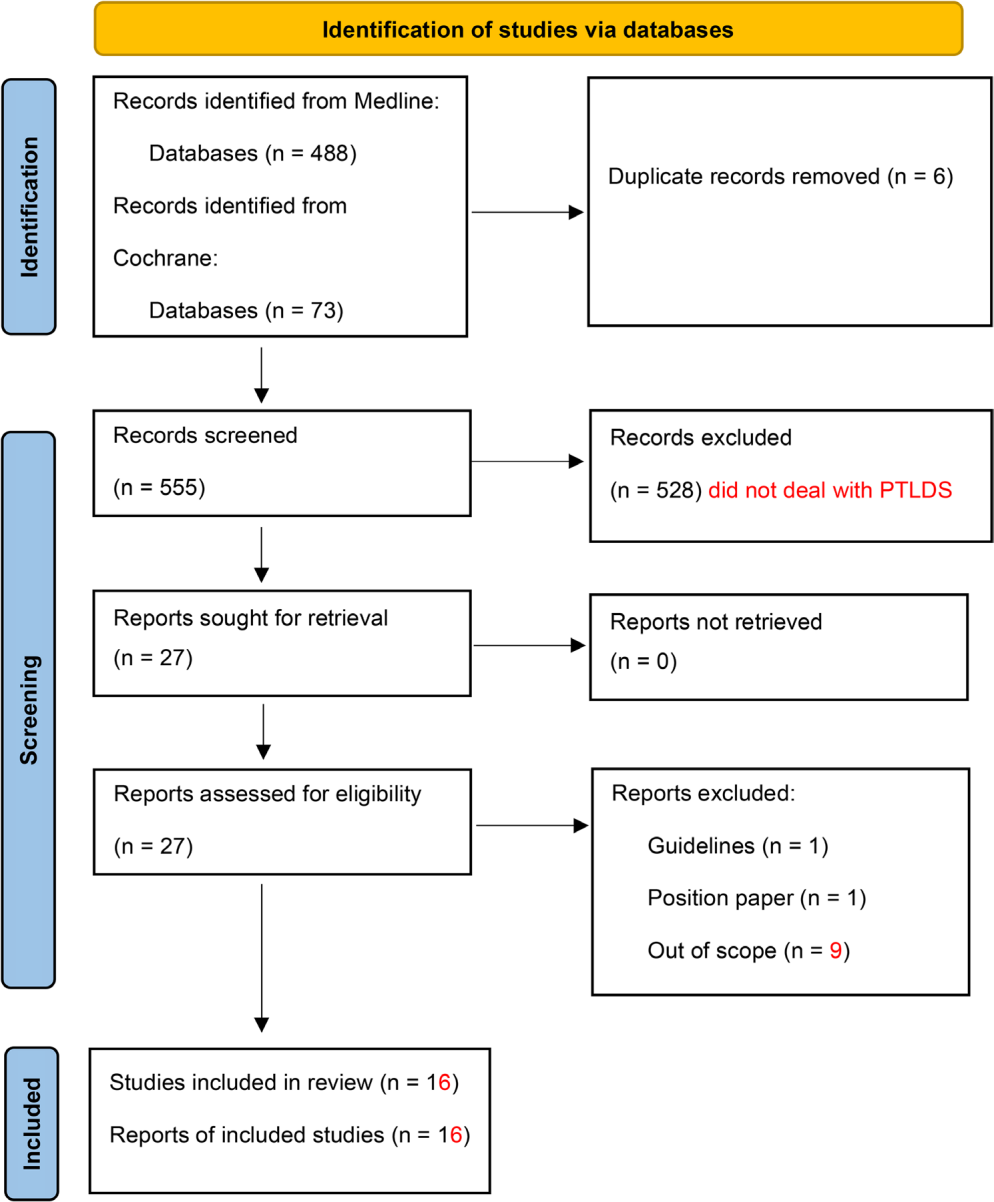
Flow diagram

The articles were selected using a quadruple reading process by SP, JD, CP and XG on the basis of the titles and abstracts of the different articles (Fig. 1). The researchers worked independently of each other. Discrepancies were discussed and resolved by consensus.

### Data analysis

For each selected article, the name of the lead author, the country, the date of publication, the method and the diagnostic errors and adverse events of the drugs used were described.

## Results

Of the 561 articles identified, 17 were included: 1 randomized clinical trial (RCT) [15], 6 observational studies [10–12, 19–21] and 10 case reports [22–31] (Fig. 1). Five hundred and twenty-eight articles were excluded, as they did not address the PTLDS. The selection process is detailed in Fig. 1. Tables 1 and 2 detail the lead author, year of publication, country of research, population, and diagnostic error-delay and adverse events.

**Table 1:** Observational and case report studies related to overdiagnosis

Type of study	Lead author Year Reference	Country	Population	Overdiagnosis
**Observational studies**	Haddad E (2019) [10] Haddad E (2019) [11] Itani O (2020) [12]	France France France	301 patients PTLDS 4 weeks 1 center Male: 60,8% Median age: 50 12–85 years old 1000 patients 3 centers Male: 50% Nancy: Median age: 51 7–86 years old 15 patients 6 months Male: 4/15 Median age: 44 15–89 years old	Overdiagnosis = 80.7% (n = 243) differential diagnosis: psychiatric (depression, post-traumatic stress, burnout syndromes, etc.) 25.2% (n = 76 ) rheumatological (osteoarthritis, scoliosis) 15.9% (n = 48) neurological (Parkinson and amyotrophic lateral sclerosis)12.3% (n = 37) OSA 4.9% (n = 15) No diagnosis 6.6% (n = 20) Overdiagnosis: 90.4, 88, 85% differential diagnoses: psychiatric 25, 19 and 13% rheumatological 16, 14 and 32% neurological 12, 6 and 5% no diagnosis 6, 29 and 26% Overdiagnosis: 100% (n = 15) differential diagnosis: psychiatric FSS 60% (n =9) neurological 20% (n =3), 1 OSA
	Kobayashi Y (2019) [20]	United States	1261 patients Male 39,2% > 12 years old Median age: 46,7	Overdiagnosis: 84.1% (n = 1061)
**Case report**	Peri F (2019) [22]	Italy	7 children Male: 3 12–17 years old	Overdiagnosis: 100% (n = 7) differential diagnosis: psychiatric (n = 6) viral infection (n = 1-
	Strizova Z (2018) [23]	Czech Republic	a 37-year-old Female systemic lupus erythematosus	Attribution to Lyme long-term tetracycline Death of multi-organ failure
	Nelson C (2015) [25]	United States	3 patients PTLDS Male: 3 30-30-50 years old ATX treatment (tetracyclines, clarithromycin and hydroxychloroquine)	diagnostic delay pituitary tumor Hodgkin’s lymphoma stage 4 lung cancer
	Di Battista ME (2018) [26]	Italy	61-year-old woman patient: doxycycline (21 days and 14 days)	diagnostic delay (4 years) frontotemporal dementia

OSA: Obstructive Sleep Apnea ; ATX: antibiotics ; LB: Lyme borreliosis ; FSS: functional somatic syndrome

**Table 2:** RCTs, observational studies and case reports related to adverse events (AEs)

RCT	Krupp LB (2003) [15]	United States	55 PTLDS patients 28 ceftriaxone IV Female 15/28 Median age 48 27 placebo Female 14/27 Median age 47 6 months	AEs: diarrhea ceftriaxone group 43% placebo group 25% Hospitalizations: 4 ceftriaxone group: 1 anaphylaxis, minor anaphylactic reactions: 2 placebo group: 3 IV catheter sepsis
**Observational studies**	Itani O (2020) [12]	France	15 patients Median age 44 15–89 years old Male: 4/15 On average 6.8 ABX / 476 days	AEs: 27% (n = 4) 1 *clostridium difficile* colitis 3 fungal infections
	Trautmann A (2020) [19]	France	16 Disulfiram patients 21–70 years old Gender not available	Various and moderate AEs: 81.2% (n = 13)
	Goodlet KJ (2018) [21]	United States	3127 patients Age and gender not available Group 1: 1102 ABX *per os* Group 2: 150 IV Groupe 3: 1875 placebo	AEs: group 1: 18.7%, group 2: 16.8%, group 3: 13.4% Infections 20.4%, gastrointestinal disorders 6.2%, electrolyte disorders 2.6% Hospitalizations: group 1: 2.2%, group 2: 7.3%, group 3: 0.9% Emergencies: group 1: 3.4%, group 2: 11.3%, group 3: 1.9%
**Cases report**	Johnstone T (2018) [27]	Australia	41-year-old woman Glutathion + Phosphatidylcholine	AEs: bacterial septicemia then *Clostridium Difficile* colitis
	Issacs D (2016) [28]	Australia	15-year-old woman Hyperthermia and ABX IV	AEs: severe dehydration due to *Clostridium Difficile* diarrhea
	Shelton A (2019) [29]	United States	32-year-old woman ABX IV (rifabutin, metronidazole, ivermectin, and pyrimethamine) then ABX *per os* (meropenem, clindamycin, tigecycline, and ciprofloxacin)	AEs: *Mycobacterium goodii* multifocal pneumonia on central venous catheter
	Marcks CM (2016) [30]	United States	45-year-old woman ABX 3 months *per os* (doxycycline, minocycline and trimethoprim-sulfamethoxazole	AEs: DRESS Syndrome
	De Wilde M (2017) [31]	Belgium	76-year-old woman ceftriaxone IV	AEs: drug-induced immunohemolytic anemia
	Patel R (2000) [20]	United States	30-year-old woman Cefotaxime IV	AEs: large Candida parapsilosis septic thrombus

Randomized Clinical Trial: RCT ; intravenous (IV) : antibiotics: ABX

### Overdiagnosis: attribution of symptoms to LB

Overdiagnosis has been described in cohort studies and case reports. The results are summarized in Table 1.

### Cohort studies

In France, two observational studies were conducted by Haddad et al. and published in 2019 [10, 11]. Rechallenging the PTLDS led to an overdiagnosis rate of 80.7%. In the second study, the overdiagnosis rate ranged from 85 to 90.4%. The differential diagnoses made were mostly psychiatric, rheumatological and neurological disorders. The categorization of differential diagnoses could be difficult, particularly for disorders with somatic symptoms (e.g. fibromyalgia), which could be classified as psychiatric, rheumatologic, or no diagnosis [11]. Another observational study published in 2020 by Itani et al. included 15 patients with PTLDS for at least six months. The overdiagnosis rate was 100% [12].

In these three observational studies, all symptomatic patients who consulted with chronic symptoms associated with Lyme disease were included. Overdiagnosis was defined as making another diagnosis using a holistic approach. A holistic approach involved a comprehensive approach to the patient, evaluating the history of presumed Lyme disease symptoms, the personal medical history, past antimicrobial treatments, all symptoms and signs, laboratory test results, and any other exams. The authors describe this holistic approach as a limitation of their studies. It is very specific needing background knowledge or interest in psychology and long consultation (30–60 min). This method was therefore difficult to generalize [10–12].

In the United States, a retrospective observational study conducted by Kobayashi et al. was published in 2019. The overdiagnosis rate was 84.1% [20]. They used established clinical and serological criteria and divided patients into 4 groups: (i) patients without Lyme disease, (ii) patients with active or recent Lyme disease including PTLDS, (iii) patients with remote Lyme disease, and (iv) patients with possible Lyme disease. Patients without Lyme disease had no clinical findings or laboratory evidence of Lyme disease. Patients with remote Lyme disease had symptoms that had started at least 2 years after complete recovery from an earlier episode of Lyme disease. Patients who were identified as over diagnosed included those who did not have Lyme disease at all and those who had a previous but distant history of Lyme disease, referred to as “patients without current Lyme disease.“ The authors describe the judgments made by the infectious disease clinicians as a limitation. They may have influenced the results, as the retrospective data collected were heterogeneous in nature [20].

### Case report

In 2015 Nelson et al. reported three cases in the United States of oncologic diagnostic errors and delays due to a diagnosis of PTLDS [25]. The first case was a 30-year-old man who had been suffering with joint pain and memory loss for 12 years. Following the onset of visual field deficit and syncopal episodes, he was diagnosed with a pituitary tumor. Facial morphological sequelae and cardiomyopathy appeared to be attributable to this diagnostic delay. The second case was a 30-year-old man with fatigue, loose stools and abdominal pain for 4 years. The diagnosis of PTLDS was made despite the absence of clinical signs of LB and living in an endemic area. The patient had received several cycles of oral and intravenous antibiotic therapy. Following discontinuation of his treatments, a gastric biopsy of a mesenteric lymph node and a PET scan revealed stage IV Hodgkin’s lymphoma. The patient died 2 years later. The third case was a 50-year-old man with asthenia for 2 weeks and an influenza-like illness for 3 days. Doxycycline adapted to early LB was prescribed. Subsequently, an erythematous rash appeared under his left shoulder. Two more courses of doxycycline were performed with partial improvement. A diagnosis of PTLDS was made. Five months after this diagnosis, an infectious disease specialist requested a chest CT since the patient had smoked for 18 years. It confirmed the diagnosis of lung cancer.

In 2016, Di Battista described the case of a 61-year-old Italian woman with cognitive impairment [26]. Four years earlier, a diagnosis of LB had been made on the basis of a typical erythema migrans. In view of cognitive disorders and a major depressive syndrome persisting despite two courses of doxycycline, PTLDS was diagnosed. A PET scan and a brain MRI were performed after one year, due to the loss of autonomy and worsening of the disorders leading to the diagnosis of frontotemporal dementia.

In 2018, Strizova et al. described the case of a 37-year-old Czech woman with lupus who attributed her symptoms to Lyme disease on the basis of her findings on the internet. She was given long-term tetracycline treatment. She died of multi-organ failure [23].

In 2019, Peri and al. analyzed medical records of 7 children with PTLDS. PTLDS had strongly influenced their schooling. A review of the clinical history revealed a 100% overdiagnosis rate [22].

### Adverse events of the drugs used

Adverse events were described in one randomized clinical trial (RCT) [15], three observational studies [12, 19, 21] and six case reports [24, 27–31].

### RCT and observational studies

In 2003, Krupp et al. conducted a randomized clinical trial with the aim of determining whether the symptoms of PTLDS regressed under antibiotic therapy [15]. The 55 patients included were randomized to receive 28 days of parenteral ceftriaxone or placebo. Diarrhea, the primary adverse event, was more common in the ceftriaxone group than placebo. Four serious adverse events required hospitalization.

In the French observational study by Itani and al. the 15 patients had received an average of 6.8 antibiotics for 476 days. Adverse events were reported in 4 patients [12].

In France, in 2020, Trautmman et al. analyzed the results of a survey sent to 3 French associations of patients with PTLDS who had taken Disulfiram [19]. Of the 16 patients who responded, 13 had experienced various and moderate side effects (headaches, dizziness, difficulty concentrating, etc.).

In the United States, in 2018, Goodlet et al. analyzed adverse reactions to oral or IV therapy in patients with PTLDS for more than 6 months [21]. The incidence rates of adverse events were higher in the IV therapy group and there were more hospitalizations.

Of these four studies, two were designed to collect adverse events related to treatments used in PTLDS [19, 21]. One study aimed to determine whether PTLDS was antibiotic responsive as assessed by clinical improvement in severe fatigue, improvement in cognitive speed, and clearance of a potential biologic marker of infection [15]. The other one aimed to determine the rate of overdiagnosis of PTLDS [12]. Adverse events were not the main objective of these two studies, which is a limitation in itself.

### Case reports

In Australia, in 2018 Johnstone et al. reported the case of a 41-year-old female patient who was treated with weekly glutathione infusions and phosphatidylcholine in a clinic for PTLDS [27]. The patient consulted the emergency department for bacterial sepsis.

In 2016, Issacs reported the case of a 15-year-old girl diagnosed by a general practitioner specializing in LB on the basis of serology performed in a private laboratory [28]. She suffered from chronic fatigue and was treated with 2 weeks of induced hyperthermia and intravenous antibiotics. These therapeutics induced severe dehydration due to *Clostridium difficile* colitis.

In the United States, in 2019, Shelton et al. reported the case of a 32-year-old woman presenting to the emergency department with fever, confusion, and dyspnea [29]. For the past two years and a diagnosis of PTLDS, she had been treated with multiple oral anti-infectives. The emergency department diagnosed multifocal pneumonia following infection of her central venous catheter with *Mycoplasma goodii*. Catheter removal and parenteral and then oral antibiotic therapy resulted in clinical improvement.

In 2016, Marks et al. reported the case of a 45-year-old woman presenting to the emergency department with a pruritic, diffuse rash with nausea and fever [30]. Six months prior to her emergency visit she had been diagnosed with PTLDS with babesiosis. She had received multiple antibiotics over the past 3 months. Emergency department blood tests showed neither active Lyme disease nor babesiosis, but a DRESS syndrome. Her condition improved with corticosteroids.

In Belgium, in 2016, De Wilde et al. reported the case of a 76-year-old woman who consulted the emergency department for malaise, vomiting, anorexia and dyspnea [31]. In 2007, she had experienced facial paresis four weeks after the onset of erythema. In 2009, a private clinic diagnosed PTLDS. She was treated for 20 consecutive weeks with 4 g of ceftriaxone IV per day. A few years later, faced with a recurrence of symptoms, the doctors proposed eight weeks of treatment. Three weeks after the start of this treatment, the emergency department diagnosed ceftriaxone-induced immunohemolytic anemia. Discontinuation of the antibiotic resulted in improvement.

In 2000, Patel described the case of a 30-year-old woman who died of nosocomial sepsis with a catheter that had been used for 27 months for treatment with ceftriaxone [24].

## Discussions

This review urges physicians to be cautious about the diagnosis of PTLDS because of the very frequent overdiagnosis which can lead to unnecessary treatments, exposing patients to potential risks and side effects. Furthermore, it can delay the identification of alternative diagnoses, leading to prolonged suffering and a missed opportunity for appropriate management.

False positive tests and non-recognized tests performed in private laboratories contribute to overdiagnosis [7, 32]. Numerous studies have highlighted a high prevalence of false positive tests and unrecognized tests performed in private laboratories, exacerbating the issue of overdiagnosis. For example, Weber et al. obtained the results of all Lyme disease serological tests ordered at U.S. Air Force healthcare facilities between January 2013 and December 2017. They conducted chart reviews to adjudicate positive IgM immunoblots (from two tiers and independent testing) as true positives or false positives using established criteria. Among 212 positive IgM immunoblot cases assessed, 113/212 (53.3%) were determined to be false positives. Antibiotics were prescribed for Lyme disease in 91/113 (80.5%) participants with a false-positive test [32]. Serologies have their limits and pitfalls, with cross-reactions, false positives, a negative serological window at the start of infection, and serological scars with suspected reinfection. Unconventional diagnostic tests have recently been developed in the context of a highly controversial and publicized disease. Raffetin et al. (2020) carried out a systematic literature review which analyzed the available data on these unconventional diagnostics. Forty studies were included: two meta-analyses, 25 prospective controlled studies, five prospective uncontrolled studies, six retrospective controlled studies and two case reports. They classified biological tests as: (i) proven to be effective at diagnosing LB and already in use (CXCL-13 for neuroborreliosis), but not enough to be standardized; (ii) not yet used routinely, requiring further clinical evaluation (CCL-19, OspA and interferon-α); (iii) uncertain LB diagnostic efficacy because of controversial results and/or poor methodological quality of studies evaluating them (lymphocyte transformation test, interferon-γ, ELISPOT); (iv) unacceptably low sensitivity and/or specificity (CD57 + natural killer cells and rapid diagnostic tests); and (v) possible only for research purposes (microscopy and xenodiagnoses) [33]. Tests with inadequate performance should not be used in routine practice, as this may expose patients to overdiagnosis and overtreatment with potential adverse events. This is also associated with delays in diagnosing neurological, psychiatric, and rheumatological conditions, which can adversely affect patient care. Further research in this domain warrants exploration. Consequently, the ACR recommends not to test for Lyme disease as a cause of musculoskeletal symptoms without an exposure history and appropriate exam findings. This recommendation underscores the importance of the diagnostic tripod: exposure to ticks, compatible signs, and positive serology [14].

The most common adverse events associated with the treatments for PTLDS were diarrhea, sometimes with *Clostridium difficile* colitis, electrolyte abnormalities, sepsis, bacterial and fungal infections, and anaphylactic reactions. These adverse events were more frequent when the anti-infectives were administered by the IV route [13, 16, 23]. The adverse events can significantly impact patients’ quality of life. In severe cases, hospitalization may be required, increasing healthcare costs and exposing patients to further risks. Moreover, the long-term consequences of these adverse events, such as gastrointestinal complications or the development of antibiotic resistance, should be carefully considered. Antibiotics are not the only drugs used without proof of efficacy. Disulfiram, as an example, has been used as an off-label treatment for PTLDS, lacking data on efficacy while being associated with significant risks and side effects.

### Strengths and limitations

To our knowledge, this literature review is the first to compile errors, diagnostic delays and adverse events associated with the diagnosis and treatments of PTLDS. While this literature review provides valuable insights into errors, diagnostic delays, and adverse events associated with the diagnosis and treatment of PTLDS, it is important to acknowledge several limitations. One limitation is the general underreporting of adverse events by caregivers and patients, which may have resulted in an underestimation of the true frequency and severity of these events. Additionally, the potential for publication bias in the included studies may have limited the completeness of our findings, as studies reporting negative outcomes or less favorable results are less likely to be published [34]. This work may allow physicians managing patients with PTLDS to report past adverse events and publish diagnostic errors and delays due to attribution of symptoms to LB.

## Conclusion

Our review suggests that PTLDS may be an over-diagnosed condition due to the use of non-standardized and non-recommended diagnostic methods in combination with a lack of adherence to diagnostic criteria. Overdiagnosis leads to over-treatment which may be associated with significant adverse events and delays in diagnosing diseases with high morbidity, such as psychiatric, rheumatological and neurological conditions. Due to the lack of a treatment gold standard, the use of prolonged antibacterials and off-label therapies may lead to adverse events without any evidence of benefit. This should raise awareness and ethical questions (*primum non nocere)* of whether healthcare providers should offer to test for Lyme disease when there is a low pretest probability of PTLDS and consider the potential risks before offering treatments with unproven efficacy.
